# High-Throughput Sequencing Enables Rapid Analyses of Nematode Mitochondrial Genomes from an Environmental Sample

**DOI:** 10.3390/pathogens14030234

**Published:** 2025-02-27

**Authors:** Akshita Jain, Tongda Li, John Wainer, Jacqueline Edwards, Brendan C. Rodoni, Timothy I. Sawbridge

**Affiliations:** 1 School of Applied Systems Biology, La Trobe University, Bundoora, VIC 3083, Australia; jackyedwards@live.com (J.E.); brendan.rodoni@agriculture.vic.gov.au (B.C.R.); tim.sawbridge@agriculture.vic.gov.au (T.I.S.); 2 AgriBio, Centre for AgriBioscience, Agriculture Victoria Research, Department of Energy, Environment and Climate Action (DEECA), Bundoora, VIC 3083, Australia; tongda.li@agriculture.vic.gov.au (T.L.); john.wainer@agriculture.vic.gov.au (J.W.)

**Keywords:** *Heterodera*, high-throughput sequencing, mitochondrial genomes, range extension, Australian biosecurity

## Abstract

Mitochondrial genomes serve as essential tools in evolutionary biology, phylogenetics, and population genetics due to their maternal inheritance, lack of recombination, and conserved structure. Traditional morphological methods for identifying nematodes are often insufficient for distinguishing cryptic species complexes. This study highlights recent advancements in nematode mitochondrial genome research, particularly the impact of long-read sequencing technologies such as Oxford Nanopore. These technologies have facilitated the assembly of mitochondrial genomes from mixed soil samples, overcoming challenges associated with designing specific primers for long PCR amplification across different groups of parasitic nematodes. In this study, we successfully recovered and assembled eleven nematode mitochondrial genomes using long-read sequencing, including those of two plant-parasitic nematode species. Notably, we detected *Heterodera cruciferae* in Victoria, expanding its known geographic range within Australia. Additionally, short-read sequencing data from a previous draft genome study revealed the presence of the mitochondrial genome of *Heterodera filipjevi*. Comparative analyses of *Heterodera* mitogenomes revealed conserved protein-coding genes essential for oxidative phosphorylation, as well as gene rearrangements and variations in transfer RNA placement, which may reflect adaptations to parasitic lifestyles. The consistently high A+T content and strand asymmetry observed across species align with trends reported in related genera. This study demonstrates the utility of long-read sequencing for identifying coexisting nematode species in agricultural fields, providing a rapid, accurate, and comprehensive alternative to traditional diagnostic methods. By incorporating non-target endemic species into public databases, this approach enhances biodiversity records and informs biosecurity strategies. These findings reinforce the potential of mitochondrial genomics to strengthen Australia’s as well as the global biosecurity framework against plant-parasitic nematode threats.

## 1. Introduction

Building accurate phylogenies to understand the nematode phylum is important, as it is estimated to include over a million species [1,2]. Nematodes are found in most environments, from scorching deserts to icy polar regions, thriving in both terrestrial and aquatic ecosystems worldwide [3,4]. They exhibit remarkable ecological breadth, including free-living species and parasitic forms that infect vertebrates, invertebrates, and plants [5]. Phylogenetic analyses of nematodes offers important insights into their evolution and diversification, revealing how their adaptations have enabled them to occupy such a wide range of ecological niches [3].

Molecular techniques have revolutionised the study of plant-parasitic nematodes (PPNs), enabling faster, more accurate identification of these pests and providing deeper insights into their biology [6]. Researchers often focus on specific ribosomal RNA segments, such as the large ribosomal subunit (28S), the partial small ribosomal subunit (18S), and the internal transcribed spacer (ITS), to explore the evolutionary relationships among different PPNs [6,7,8,9,10,11,12,13]. However, to provide greater phylogenetic resolution, more powerful genetic markers are needed. Complete mitochondrial genomes (mitogenomes) have emerged as a promising option, offering significantly improved accuracy in determining evolutionary relationships when compared to gene-based phylogenetic comparisons [14]. Mitogenomes also enable a more precise estimation of divergence times between species [15] and are increasingly used to resolve taxonomic disagreements [16,17,18,19,20,21,22,23,24]. Mitogenomes offer several key advantages that enhance their effectiveness, including their manageable genome size, which allows for efficient analysis on standard computer hardware [14], and the encoding of a conserved set of genes across metazoans that enable the identification of unambiguous orthologs (genes with a common evolutionary origin) between nematode species. Mitogenome sequence analyses can identify variations in gene order, which can be used as supplementary evidence for phylogenetic relationships [25] and can reflect evolutionary events that provide valuable insights into lineage divergence [4]. Mitochondrial DNA (mtDNA) evolves at a faster rate than nuclear DNA (nDNA), resulting in higher sequence variation and providing a finer resolution for distinguishing closely related species [26,27,28]. This characteristic makes mitogenomes a promising source of genetic markers for distinguishing closely related taxonomic groups. Single-locus markers within the mitogenome, such as the protein-coding genes (PCGs) cytochrome c oxidase subunit I (COX1) and NADH dehydrogenase subunit 1 (ND1), evolve rapidly and can capture species-level genetic variations showing clear distinctions between species through accumulated mutations over time [20]. Overall, nematode nuclear genomes are larger, more complex, and more difficult to assemble and annotate, with larger sequencing costs. They are also biparentally inherited, resulting in a broader genetic perspective for understanding long-term evolutionary processes. In contrast, mitogenomes are smaller, simpler, and maternally inherited, making them ideal for species identification and the study of recent evolutionary history [20,27].

Nematode mitogenomes are typically compact, haploid, single circular DNA molecules ranging from 12 to 22 Kbp in size [29]. They encode a core set of genes found in most metazoan mitogenomes, including 12–13 PCGs, including ND1, ND2, ND3, ND4, ND5, and ND6 (NADH dehydrogenase subunits 1, 2, 3, 4, 5, and 6); COX1, COX2, and COX3 (cytochrome c oxidase subunits 1, 2, and 3); CYTB (cytochrome b); ND4L (subunit 4L of NADH dehydrogenase); ATP6 (Adenosine triphosphate subunit 6); and/or ATP8 (Adenosine triphosphate subunit 8), all of which are involved in oxidative phosphorylation. Additionally, nematode mitogenomes contain 22 transfer RNA (tRNA) genes essential for protein synthesis and 2 ribosomal RNA (rRNA) genes involved in protein translation [18,26]. Nematode mitogenomes exhibit some unique features compared to other animals. One distinctive characteristic is the frequent absence of the ATP8 gene, which is present in most other metazoans [14], though exceptions exist, such as in *Trichinella* species [30,31] and *Trichuris* species [32,33,34]. Additionally, nematodes from the *Globodera* genus display a multipartite mitochondrial genome, where genetic material is distributed across several smaller circular DNA molecules, a feature not commonly seen in other metazoans [35,36,37,38,39,40]. However, given the phylum’s taxonomic richness, the current 250 or more sequenced nematode mitogenomes offer only a glimpse of nematode diversity [14,23]. Of the publicly available nematode mitogenomes, only a small number are PPN mitogenomes (Table 1).

The genus *Heterodera* includes a group of plant-parasitic nematodes, commonly referred to as cyst nematodes, which are responsible for significant economic damage to various agricultural crops. These microscopic roundworms interact with their plant hosts in a way that leads to growth retardation, wilting, and reduced yields [52]. *Heterodera* species are characterised by a distinct life cycle in which females develop into immobile cysts after penetrating plant roots. These cysts house the next generation of eggs, ensuring the survival and spread of the nematode population. Understanding the biology of *Heterodera*, particularly their unique adaptations to parasitism, is crucial for developing effective management and control strategies [53,54,55]. The Heteroderidae family within the Hoplolaimoidea superfamily offers promising potential for using mitochondrial gene rearrangements as phylogenetic markers, supported by extensive rearrangements observed across its species [44].

Despite these initial insights, significant gaps remain in our understanding of *Heterodera* evolution due to the limited availability of mitogenomic data [18]. To construct a more complete picture of *Heterodera* evolutionary history and identify potential targets for control and management strategies, additional mitogenome sequences from a range of *Heterodera* species is essential. The objective of this study was to develop an efficient workflow for extracting nematode mitogenomes from a metagenomic sequence dataset generated from a field-collected soil sample using a concentrated nematode suspension and long-read sequencing technology. A secondary goal of the study was to extract *Heterodera* mitogenomes assembled from short-read sequencing data generated during a whole-genome sequencing study of *Heterodera* species [56]. By generating mitochondrial DNA sequences for *Heterodera* species, this study contributes to a growing body of nematode genome sequence data and provides a molecular resource for future research.

## 2. Materials and Methods

### 2.1. Soil Sampling, DNA Extraction, and Long-Read Sequencing

Soil samples were collected from a broccoli growing paddock as part of a routine survey of vegetable growing blocks in the Werribee region south-west of Melbourne (Victoria, Australia). Two hundred grams of soil was placed into a tray lined with two-ply tissue paper for Whitehead tray nematode extractions. The Whitehead trays were left undisturbed for 48 h at room temperature. After the incubation period, 25 mL of concentrated nematode suspension was collected from the tray using a 20 µm sieve. To enhance extraction efficiency, the nematode solution was sieved twice. The concentrated 25 mL nematode suspension (containing free-living nematodes and a few plant-parasitic juveniles) was stored at 4 °C until DNA extraction.

The remaining wet soil was placed in a Fenwick can for cyst nematode extraction. The filter paper for the Fenwick can extraction was examined, and the presence of *Heterodera* cysts was confirmed under a microscope.

The nematode suspension was divided into 80 biological replicates of 200 µL each. Genomic DNA was extracted from all 80 replicates using the DNeasy Blood and Tissue kit (Qiagen, Hilden, Germany), following the manufacturer’s protocol. The DNA was eluted into 50 µL each and quantified using the Quantus Fluorometer (Promega, Madison, WI, USA). The eluted DNA samples (80 replicates) were pooled and concentrated using Promega Pronex^®^ size-selective beads (Promega, Madison, WI, USA) to achieve a sufficient concentration for long-read sequencing on the Oxford Nanopore MinION. A DNA concentration of 7.0 ng/µL was obtained from the concentrated nematode suspension.

Concurrently, genomic DNA was extracted from 10 randomly selected *Heterodera* cysts in individual reactions using the QIAamp Micro DNA Extraction kit (Qiagen, Hilden, Germany), following the manufacturer’s protocol. The only modification was that the cysts were mixed with the extraction buffer and proteinase K in a Thermomixer^®^ (Eppendorf©, Hamburg, Germany) and incubated overnight at 800 rpm at 56 °C. DNA was eluted in 50 µL of elution buffer and quantified using the Quantus Fluorometer (Promega, Madison, WI, USA) with a concentration of 0.173 ng/µL, and the cysts were identified using PCR followed by Sanger sequencing and analysis, as described by Jain, Wainer et al. [57]. The cysts were molecularly identified as *Heterodera schachtii*, a common plant-parasitic nematode found in *Brassica* paddocks in south-western Melbourne, Victoria, Australia.

A library for long-read metagenomic sequencing was prepared from the concentrated nematode suspension DNA. Ten microlitres of *H. schachtii* cyst genomic DNA was spiked in as a positive control, and the library was prepared using the Oxford Nanopore Technologies Ligation Sequencing DNA V14 (SQK-LSK114) kit according to the manufacturer’s instructions. The final library concentration was 7.2 ng/µL in a total volume of 48 µL and was sequenced using the MinION Flow Cell (R10 version—FLO-MINI14) over a 72 h period.

Raw sequencing data from the nematode suspension library were base-called using ONT Dorado (https://github.com/nanoporetech/dorado, accessed on 18 August 2024) with the super-high-accuracy model. Sequencing adapters, index sequences, and low-quality bases were removed during base calling. A metagenomic de novo assembly was generated using Flye [58] with the following parameters: —genome-size 50 m—threads 48—iterations 3—keep-haplotypes—read-error 0.03—meta.

### 2.2. Interrogating Draft Genomes Generated Using Short-Read Technology for Deducing Heterodera mitogenomes

Jain, Li et al. [56] sequenced the genomic DNA of six *Heterodera* species (*H. australis*, *H. avenae*, *H. filipjevi*, *H. humuli*, *H. mani*, and *H. trifolii*) using 50 cysts per species on the Illumina short-read sequencing platform. The resulting genome assembly files (GenBank BioProject number PRJNA1109461) were analysed for the presence of mitogenomes by using the approach described below.

### 2.3. Assembly and Annotation of Mitogenomes and Sequence Analysis

Mitochondrial genes were identified using a combination of methods. First, MitoFinder v1.4.2, a Linux-based software [59], was employed using the FASTA assembly file obtained from the long-read sequencing data, with a mitochondrial genome of *Heterodera glycines* (NCBI GenBank accession number HM640930) [44] serving as the reference sequence. MitoFinder performed a series of BLAST searches using the reference sequence to identify contigs containing mitochondrial DNA using the following parameters: minimum contig length = 14,000 bp, organism code = invertebrate, and nucleotide BLAST percentage identity = 70%. MitoFinder returned two separate annotated files for the two identified mitochondrial contigs, which were subsequently analysed in Geneious Prime (https://www.geneious.com/, accessed on 13 September 2024). Each of the assembled contigs represented a nematode mitogenome.

For the second method, the mitochondrial contigs identified by MitoFinder were extracted and annotated using the web-based MITOS2 pipeline [60,61] available on Galaxy [62]. The invertebrate genetic code and metazoan reference data (RefSeq63) were used for annotation. The final overlapping nucleotides were reduced to ten per gene, with an e-value threshold of 0.01. Annotations of the assembled mitogenomes from both MitoFinder and MITOS2 were then manually curated and visualised in Geneious Prime. The base content % was calculated in Geneious Prime. The nucleotide composition bias of the generated mitogenomes was calculated using the following formulae: AT bias = (A − T)/(A  +  T) and GC bias  =  (G − C)/(G  +  C) [63]. Putative secondary structures of the tRNA genes were inferred using tRNAscan-SE [64], as employed by MitoFinder, and verified using the MITOS2 output files.

### 2.4. Other Nematode Mitogenomes Extracted from the Long-Read De Novo Assembly

Contigs generated from the de novo assembly of the long-read metagenomic dataset were further analysed for the presence of any nematode mitochondrial sequences other than *Heterodera* spp. using multiple reference sequences, including *Meloidogyne arenaria* (GenBank accession number NC_026554), *Xiphinema rivesi* (GenBank accession number KU746820), *Bursaphelenchus xylophilus* (GenBank accession number NC_023208), *Aphelenchoides besseyi* (GenBank accession number NC_025291), *Pratylenchus vulnus* (GenBank accession number NC_020434), *Steinernema abbasi* (GenBank accession number MG970364), and *Steinernema carpocapsae* (GenBank accession number AY591323). MitoFinder then performed a series of BLAST searches using these reference sequences to identify contigs containing mitochondrial DNA, applying the following parameters: minimum contig length = 10,000 bp, organism code = Invertebrate, and nucleotide BLAST percentage identity = 50%.

### 2.5. Phylogenetic Analyses

Phylogenetic analyses were conducted using 44 nematode mitochondrial DNA sequences (Supplementary Table S1) using the concatenated protein-coding genes, including the ND1, ND2, ND3, ND4, ND5, and ND6 (NADH dehydrogenase subunits 1, 2, 3, 4, 5, and 6); COX1, COX2, and COX3 (cytochrome c oxidase subunits 1, 2, and 3); CYTB (cytochrome b); ND4L (subunit 4L of NADH dehydrogenase); ATP6 (ATP synthase subunit 6); and 45 COX1 gene sequences (Supplementary Table S2) obtained from NCBI GenBank, along with those identified in this study. Sequences were aligned using MAFFT [65,66] alignment (v7.450). Majority-rule consensus trees were constructed using maximum likelihood (ML) analyses. ML analyses were performed using RAxML v8 [67], employing 1000 bootstraps replicates, the GTR Gamma I nucleotide model, rapid bootstrapping, and a search for the best-scoring ML tree algorithm. The resulting ML trees were combined and edited in Microsoft PowerPoint for visualisation.

## 3. Results

The MitoFinder pipeline using the *Heterodera glycines* (NCBI GenBank accession number HM640930) mitogenome assembled two distinct cyst nematode mitogenomes, *H. schachtii* and *H. cruciferae*, from the long-read assembly sequenced from the nematode suspension spiked in with genomic DNA of *H. schachtii*. The *H. schachtii* mitogenome showed an 89.49% similarity to the reference *H. glycines* sequence (NCBI GenBank accession number HM640930) [44], based on NCBI BLAST results. The second mitogenome was identified as *H. cruciferae* with a 78.04% similarity to the *H. glycine* sequence [44]. Although the library sequenced on the MinION included only *Heterodera schachtii* cysts, identified through PCR-based diagnostics, the sequencing results also revealed a distinct mitochondrial contig for *Heterodera cruciferae*.

A similar approach was used to extract the mitogenomes from the draft genome assemblies of *Heterodera* species generated through a short-read metagenomics approach [56]. Only one *Heterodera* species, *H. filipjevi*, yielded a mitogenome, with the assembled contig showing a 78.55% similarity to the *H. glycines* reference mitochondrial sequence based on NCBI BLAST results.

**Figure 1:**
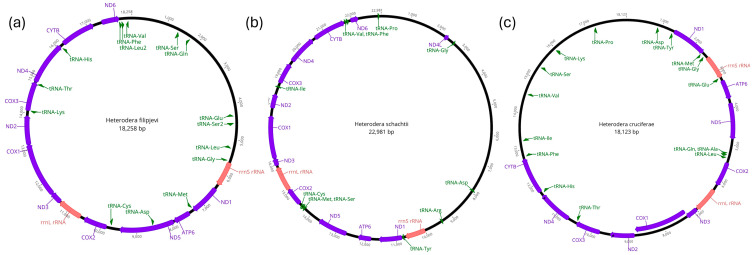
Circular mitochondrial genome maps of three *Heterodera* species extracted in this study: (**a**) *Heterodera filipjevi* (GenBank accession number PQ178946), (**b**) *Heterodera schachtii* (GenBank accession number PQ325512), and (**c**) *Heterodera cruciferae* (GenBank accession number PQ325513). These maps provide a visual representation of the mitochondrial genomes, highlighting the arrangement and orientation of various genes within the *Heterodera* species mitogenomes. The maps display both coding and non-coding regions (black). Protein-coding genes are depicted in purple, including ND1, ND2, ND3, ND4, ND5, and ND6 (NADH dehydrogenase subunits 1–6); COX1, COX2, and COX3 (cytochrome c oxidase subunits 1–3); CYTB (cytochrome b); ND4L (subunit 4L of NADH dehydrogenase); and ATP6 (Adenosine triphosphate subunit 6). Two ribosomal RNA (rRNA) genes, rrnS (12S ribosomal RNA) and rrnL (16S ribosomal RNA), are shown in red. Various tRNA genes are indicated in green, with tRNAs labelled according to the amino acids they transport. Gene annotations include their approximate nucleotide positions on the circular map, providing an overview of their locations within the genome. The direction of transcription for each gene is indicated by the arrowhead orientation on the gene blocks. Genes oriented clockwise or counterclockwise on the circular map reflect the strand they are transcribed on.

The mitogenomes of *Heterodera filipjevi* (GenBank accession number PQ178946, Figure 1a)*, H. schachtii* (GenBank accession number PQ325512, Figure 1b), and *H. cruciferae* (GenBank accession number PQ325513, Figure 1c) assembled and annotated in this study are long, circular, double-stranded DNA molecules, measuring 18,258 bp, 22,981 bp, and 18,120 bp in length, respectively, and each contains a total of 25, 28, and 29 genes, respectively. The average sequencing depth for the *H. filipjevi*, *H. schachtii*, and *H. cruciferae* mitogenomes was 6×, 7×, and 126× times, respectively. The detailed mitochondrial genome organisation for these three *Heterodera* species is shown in Table 2.

**Figure 2:**
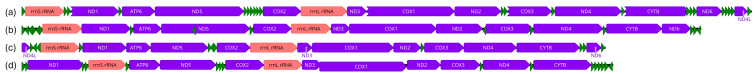
A comparison of mitochondrial gene* order across four species of *Heterodera*: (**a**) *Heterodera glycines* (NCBI GenBank accession number HM640930), (**b**) *Heterodera filipjevi* (GenBank accession number PQ178946), (**c**) *Heterodera schachtii* (GenBank accession number PQ325512), and (**d**) *Heterodera cruciferae* (GenBank accession number PQ325513). Each species’ mitochondrial genome is represented by an arrangement of coding sequences (purple), transfer RNAs (green), and ribosomal RNAs (red). The directions of the arrows represent the directions of the genes. Non-coding regions are not shown. *Gene and mitochondrial genome size are not to scale.

The *Heterodera* mitogenomes assembled and annotated in this study along with the *H. glycines* mitogenome [44] share a similar set of PCGs (Figure 2), including ND1, ND2, ND3, ND4, ND5, ND6, COX1, COX2, COX3, ATP6, and CYTB. However, there are some differences: the ND4L gene is missing in the mitogenomes of *H. filipjevi* and *H. cruciferae*, while ND6 is missing in the *H. cruciferae* genome.

The tRNA genes, shown in green (Figure 2), are scattered throughout the genomes, interspersed between the PCGs. In contrast to *H. glycines* [44], a total of 15, 11, and 16 tRNA genes were present in the *H. filipjevi*, *H. schachtii*, and *H. cruciferae* mitogenomes, respectively. The positioning and distribution of these tRNA genes differ slightly among the species, with some tRNA genes missing in the *H. filipjevi*, *H. schachtii*, and *H. cruciferae* mitogenomes. The 16S ribosomal RNA (rrnL) gene for the three species ranges from 818 bp (in *H. schachtii*) to 664 bp (in *H. cruciferae*) and is located between two PCGs, COX2 and ND3, in the forward direction (Figure 1). The 12S ribosomal RNA (rrnS) gene in the three species is positioned in different locations on the sense strand. For *H. filipjevi*, the rrnS (676 bp) is positioned between tRNA-Gly and ND1 (Figure 1). In *H. schachtii*, the rrnS gene (674 bp) is found between tRNA-Asp and tRNA-Tyr, whereas in *H. cruciferae* the rrnS gene (644 bp) is positioned between tRNA-Gly and tRNA-Glu (Figure 1).

The A+T content present in the *Heterodera* mitogenomes ranged from 80.3% to 82.7% (Table 3), with an average of 81.7%, showing a significant A/T bias. All *Heterodera* species showed significant negative AT skew and positive GC skew, further supporting the conclusion that their mitogenomes are substantially A/T-biased [23] (Table 3). In contrast, *Globodera* species had a slightly lower A+T content (65.6% to 67.0%), with *Globodera vulgaris* being an exception, displaying a positive AT skew (0.05), indicating a slight excess of adenine over thymine. Additionally, most *Globodera* species had positive GC skew values, except *G. vulgaris*, which showed a negative GC skew (−0.05), indicating a relative cytosine abundance [35].

### 3.1. Free-Living Nematode Mitochondrial Sequences Assembled from the Long-Read Data

**Figure 3:**
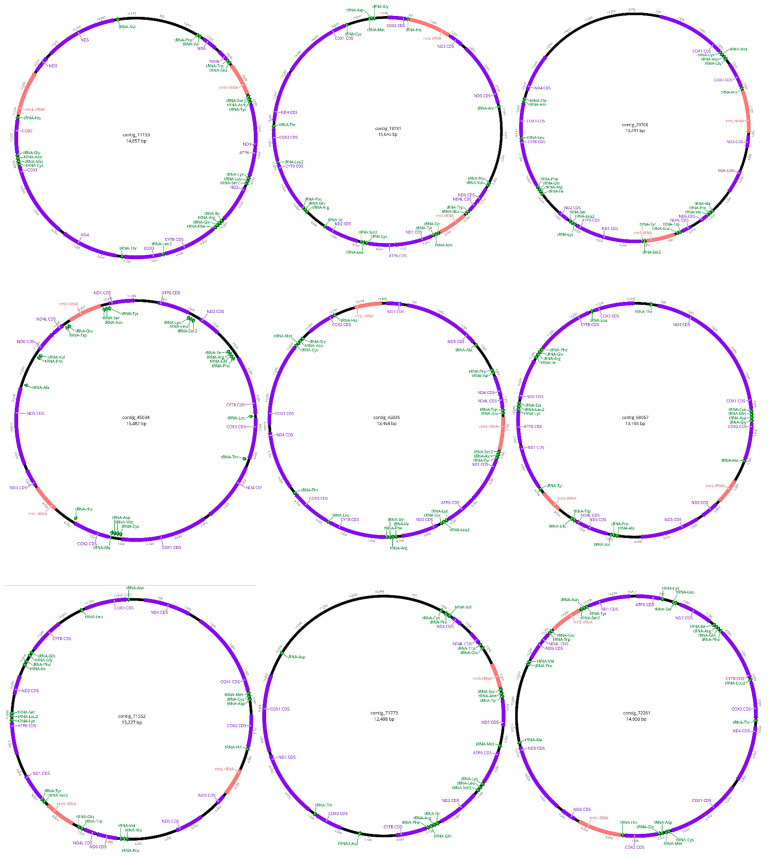
Circular mitochondrial genome maps of nine free-living nematode contigs obtained from the long-read assembly. The maps display both coding and non-coding regions (black). Protein-coding genes are depicted in purple, including ND1, ND2, ND3, ND4, ND5, and ND6 (NADH dehydrogenase subunits 1–6); COX1, COX2, and COX3 (cytochrome c oxidase subunits 1–3); CYTB (cytochrome b); ND4L (subunit 4L of NADH dehydrogenase); and ATP6 (Adenosine triphosphate subunit 6). Two rRNA genes, rrnS and rrnL, are shown in red. Various tRNA genes are indicated in green, with each tRNA labelled according to the amino acid it transports. Gene annotations include their approximate nucleotide positions on the circular map, providing an overview of their locations within the genome. The direction of transcription for each gene is indicated by the arrowhead orientation on the gene blocks. Genes oriented clockwise or counterclockwise on the circular map reflect the strand they are transcribed on.

Nine nematode mitogenomes (Table 4, Figure 3) were obtained from the long-read sequencing dataset generated from the nematode suspension using MitoFinder, showing varying GC contents (from 13.4% to 25.7%) and lengths (12,498 to 15,645 bp). The BLASTn matches primarily correspond to free-living nematode species, with nucleotide identity percentages ranging from 80.51% to 89.21% when compared to publicly available sequences on NCBI GenBank.

### 3.2. Mitochondrial Phylogeny of Nematode Mitogenomes Assembled During This Study

**Figure 4:**
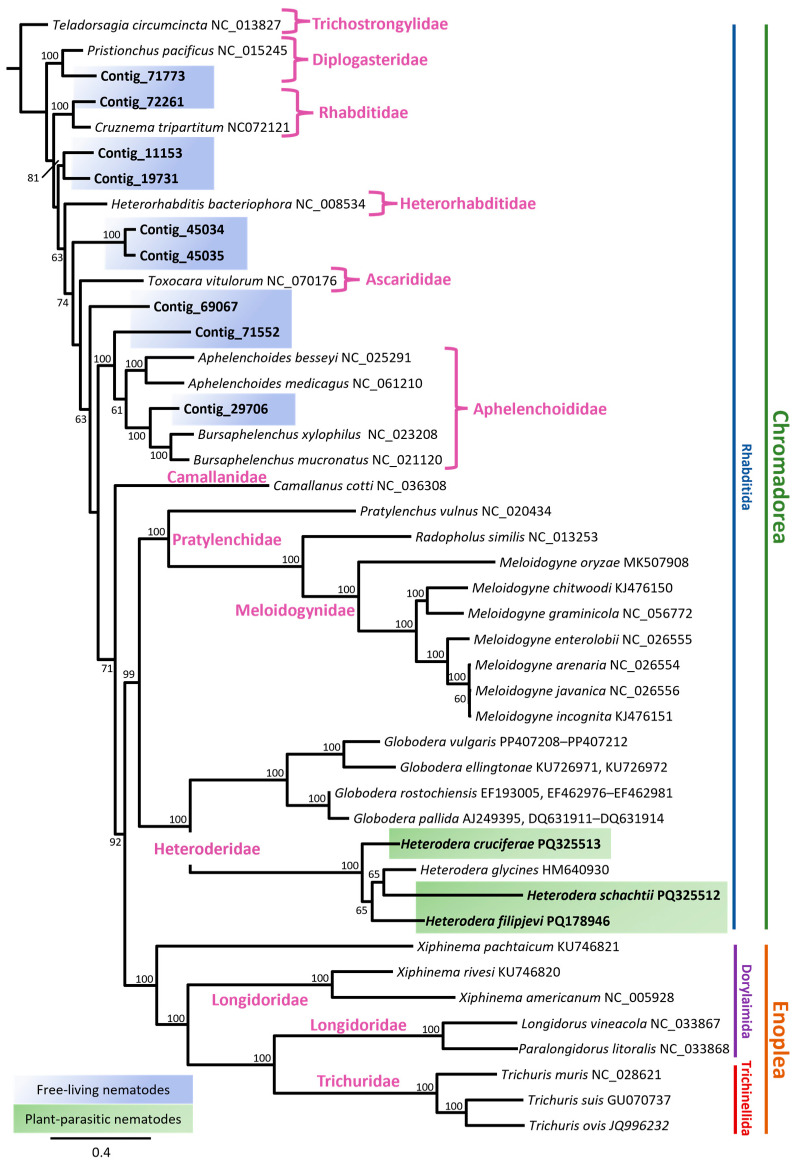
Maximum likelihood phylogenetic tree of nematodes with bootstrap values shown at internal nodes where support was 50% or greater. The tree was inferred from the nucleotide sequences of twelve concatenated mitochondrial protein-coding genes, including ND1, ND2, ND3, ND4, ND5, and ND6 (NADH dehydrogenase subunits 1–6); COX1, COX2, and COX3 (cytochrome c oxidase subunits 1–3); CYTB (cytochrome b); ND4L (subunit 4L of NADH dehydrogenase); and ATP6 (Adenosine triphosphate subunit 6) across 44 nematode mitogenomes (Supplementary Table S1), using MAFFT alignment of the concatenated protein-coding gene sequences under the GTR Gamma I model in RAxML. New sequences are highlighted in bold on the appropriate branches with their species name followed by the GenBank accession number. The scale bar represents the number of substitutions per site. Nematode mitogenomes obtained in this study were classified as free-living (blue) and plant-parasitic (green). *Teladorsagia circumcinta* (GenBank accession number NC_013827) was used as the outgroup.

**Figure 5:**
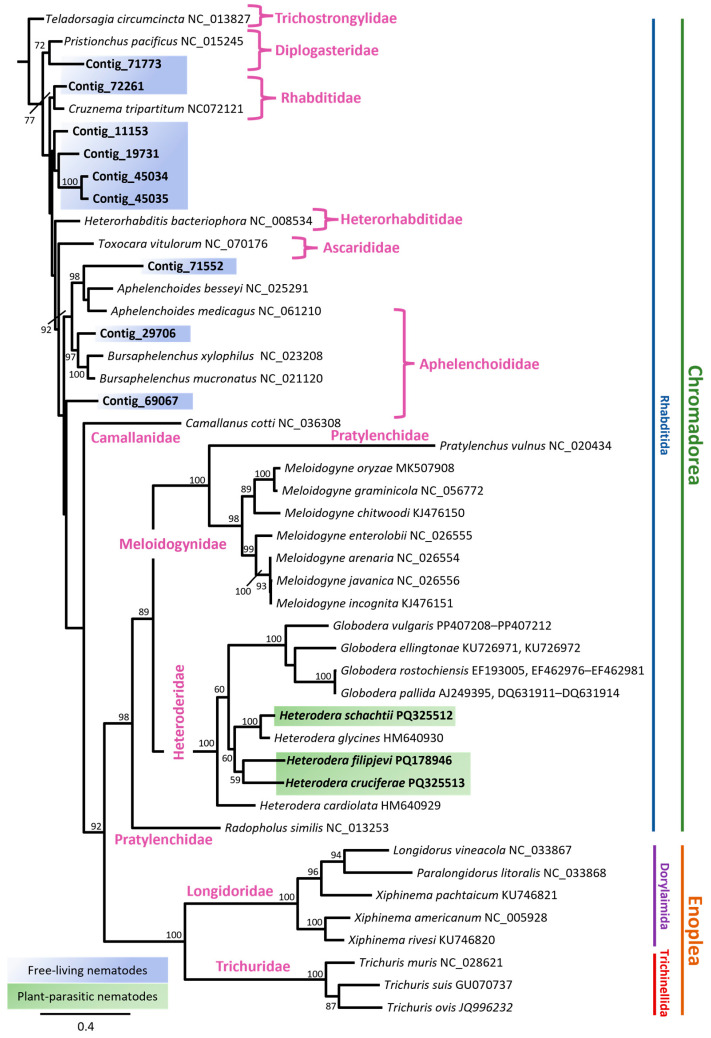
Maximum likelihood phylogenetic tree of nematodes with bootstrap values shown at internal nodes where support was 50% or greater. The tree was inferred from nucleotide sequences of the COX1 gene for 45 nematode mitogenomes (Supplementary Table S2), using MAFFT alignment of COX1 sequences under the GTR Gamma I model in RAxML. New sequences are highlighted in bold on appropriate branches with their species name followed by the GenBank accession number. The scale bar represents the number of substitutions per site. Nematode mitogenomes obtained in this study were classified as free-living (blue) and plant-parasitic (green). *Teladorsagia circumcinta* (GenBank accession number NC_013827) was used as the outgroup.

The mitochondrial phylogeny of nematodes was reconstructed using maximum likelihood (ML) analysis of complete or nearly complete mitogenomes (Supplementary Tables S1 and S2). The resulting phylogenetic tree was generated through ML analyses of nucleotide sequence datasets for 12 concatenated protein-coding genes (PCGs), including ND1, ND2, ND3, ND4, ND5, and ND6; COX1, COX2, and COX3; CYTB; ND4L; and ATP6, as well as the COX1 gene, and revealed clear clustering of major nematode lineages. Phylogenetic relationships were mainly consistent with previous reports based on mitochondrial genome analysis [16,22,68,69,70]. For example, the placement of the Pratylenchidae family in the PCG sequence dataset was not well-resolved (Figure 4). The positions of species within the family were, however, well supported in the COX1 phylogenetic tree (Figure 5).

The maximum likelihood phylogeny (Figure 4 and Figure 5) revealed distinct clades corresponding to nematode taxa with different ecological niches. Chromadoria, the most diverse group of nematodes, included multiple subclades forming monophyletic branches with strong support in both analyses. The plant-parasitic cyst nematodes (*Globodera* and *Heterodera* spp.) formed a well-supported monophyletic group (bootstrap value = 100). Within this clade, *Globodera pallida* and *G. rostochiensis* clustered together, while *G. ellingtonae* and *G. vulgaris* formed a separate but closely related subclade (Figure 4). *H. cruciferae* and *H. filipjevi* showed polyphyletic relationships with both *H. schachtii* and *H. glycines*. The *H. glycines* and *H. schachtii* clade was strongly supported with a high bootstrap value (Figure 5).

Adjacent to the cyst nematodes, other plant-parasitic nematodes, including *Meloidogyne* spp. and *Pratylenchus vulnus*, exhibited strong bootstrap support (bootstrap values > 90). In the family Longidoridae, two well-supported subclades were identified in both phylogenies (Figure 4 and Figure 5). One clade included *Longidorus*, *Paralongidorus*, and *Xiphinema pachtaicum*, with strong support in the ML analysis; however, *X. pachtaicum* formed a monophyletic branch and was not clearly associated with any specific subclade. The second subclade comprised the remaining *Xiphinema* species, with *Xiphinema americanum* and *X. rivesi* showing a close relationship, strongly supported by the analysis (100% bootstrap support).

Contig_71773 formed a very well-supported monophyletic relationship with *Pristionchus pacifus* (Figure 4) in the concatenated PCG phylogeny, but the support in the COX1 phylogeny was weaker (support value = 72%) (Figure 5). Both Contig_69067 and Contig_29706 formed well-supported branches within the Aphelenchoididae family. While both contigs formed monophyletic groups, it remains unclear whether they belong to the same genus or closely related genera. Similarly, contigs 45034, 45035, 11153, and 19731 formed well-supported clades; however, their taxonomic identification remains unresolved.

## 4. Discussion

A total of 12 mitogenomes were assembled from both parasitic (Figure 1) and free-living nematode genomes (Figure 3) sequenced using long- and short-read sequencing techniques. While 11 mitochondrial sequences were obtained using long-read assembly, only one was obtained from the short-read data (GenBank BioProject number PRJNA1109461), sequenced on the Illumina NovaSeq platform [56].

Nematode mitochondrial genomes, being relatively small, circular, and with a conserved gene order, provide an ideal target for molecular detection and identification and evolutionary studies [14]. A major bottleneck in previous nematode mitogenome sequencing studies was the difficulty in primer selection for long PCR amplification [71]. However, the advent of short- and long-read high-throughput sequencing (HTS) technologies has overcome this limitation, enabling the extraction of near-complete mitogenomes [71]. This method has been successfully applied to a limited number of nematode species, including *Meloidogyne graminicola* [48], *Globodera ellingtonae* [37], *Hoplolaimus columbus* [72], *Aphelenchoides medicagus* [42], and *Cruznema tripartitum* [71].

The successful recovery and assembly of eleven nematode mitogenomes, including two PPN genomes, using MinION (long-read sequencing technology) in this study emphasises the significant technological advancements in HTS enabling rapid identification of (nearly) complete nematode mitogenomes, allowing them to be used for diagnostic purposes and surveillance strategies. The identification of two distinct cyst nematode mitogenomes, one unexpected based on initial PCR-based cyst identification, from a single de novo long-read assembly illustrates the capability of long-read sequencing technology to resolve complex nematode populations within the same genus from field samples. While the average coverage for *H. schachtii* was extremely low when compared to *H. cruciferae*, nearly complete mitogenomes were obtained.

The mitochondrial genome of *H. schachtii* reported in this study is notably larger than those of closely related PPNs, such as *Radopholus similis* (16,791 bp, NCBI GenBank accession number NC_013253) [49] and *Pratylenchus vulnus* (21,656 bp, NCBI GenBank accession number GQ332425) [22]. This finding aligns with earlier estimates of *H. schachtii* mitochondrial genome size derived from restriction fragment studies [15].

Although the long-read library was spiked with *H. schachtii* cysts, the identification of the *H. cruciferae* mitogenome was an important detection, since this species had previously only been reported in South Australia, Australia [73,74], and not in Victoria, Australia. *H. cruciferae* was first reported in Australia around 50 years ago, in South Australia, and may have existed in Victoria since then. However, due to potential misidentification, the presence of *H. cruciferae* could have been overlooked similarly to the previously unknown presence of *H. daverti* in Victoria due to its close resemblance to *H. trifolii* [57]. Detection of *H. cruciferae* has important agricultural implications, especially considering that Victoria produced approximately 35,000 tons of fresh broccoli and baby broccoli in 2022/23, accounting for 47% of Australia’s total production for export, processing, and domestic supply [75]. As *H. cruciferae* is a cyst nematode species known to infect all *Brassica* species [53,76], its presence in Victoria could significantly impact local agricultural industries.

This workflow also enabled the characterisation of non-target, free-living nematode species (Figure 3), contributing to the enrichment of public databases and preserving biodiversity data that are often overlooked or discarded. Long-read sequencing technology offers significant advantages in assembling genomes with greater accuracy and completeness, particularly for complex or previously uncharacterised organisms [77,78]. Notably, some Oxford Nanopore reads can be nearly as long as an entire mitochondrial sequence, reducing the need for excessively high sequencing coverage and depth [79,80]. Low-coverage, long-read sequencing, as employed for the identification of individual root-knot nematodes, provides an alternative approach for the assembling of genomes [80]. In diagnostic applications, instead of focusing primarily on complete genome assemblies, this approach can prioritise taxonomic identification, the discovery of novel genetic variability, and the characterisation of virulence-related loci [80]. Furthermore, the sequencing of highly AT-rich regions, common in nematodes, is unbiased [81], a feature particularly beneficial in metagenomic environmental samples like those used in this study, where multiple AT-rich nematode species, both target (Table 3) and non-target (Table 4), may be present.

Oxford Nanopore technology (ONT) enabled the identification of multiple cyst nematode species co-existing within the same agricultural field which may be morphologically indistinguishable by the naked eye [76]. This highlights the complexity of nematode communities in agricultural soils and the potential for missed diagnoses when relying solely on morphology-based techniques, which are labour-intensive and time-consuming [82]. Additionally, investigation of short-read data from a *Heterodera* draft genome study by Jain, Li et al. [56] revealed only a single mitogenome from *Heterodera filipjevi*. These data generated using Illumina short-read sequencing were derived from an S1 NovaSeq flowcell with a 150 bp read length for five species—*H. australis*, *H. avenae*, *H. humuli*, *H. mani*, and *H. trifolii*—while *H. filipjevi* was sequenced on an SP flowcell with a 250 bp read length. We speculate that this difference of a longer read length may explain why only a single long contig containing the mitochondrial DNA sequence of *H. filipjevi* was obtained [83].

The mitogenomes of the three *Heterodera* species (Figure 1) analysed in this study share a highly conserved set of PCGs involved in oxidative phosphorylation, a critical pathway for cellular respiration and energy production [14]. The absence of ND4L in both *H. filipjevi* and *H. cruciferae* and the absence of ND6 in *H. cruciferae* suggest species-specific variations that may reflect adaptations to their metabolic or parasitic stages [23]. The absence could also highlight the delimiting nature of gene recovery in a nematode suspension sample. Whilst this is just speculative, future research is needed to understand whether the absence/presence of certain PCGs is linked to parasitic lifestyle adaptations or not. Gene rearrangements, which are commonly observed in nematode mitogenomes, were evident in the slight differences in gene order across the species (Figure 2). These rearrangements are likely driven by genomic events, such as inversions, transpositions, and duplications [21].

The presence of scattered tRNA genes interspersed between PCGs in the mitogenomes of *H. schachtii*, *H. filipjevi*, and *H. cruciferae* (Figure 1) further highlights the flexible architecture of nematode mitogenomes [23]. Despite this variability, tRNA genes play an essential role in mitochondrial protein synthesis, and their precise placement could potentially influence the efficiency of protein translation. This suggests that the placement of tRNA genes may contribute to specific adaptations within these species [84]. The observed differences in tRNA gene placement hint at the plasticity of the mitochondrial genome, which can lead to genetic diversity through structural changes while maintaining conserved functions [23]. This mitochondrial plasticity was also observed in *Meloidogyne* species, where the movement of tRNA genes likely occurred multiple times within the genus, possibly originating from a common ancestor, such as a *Pratylenchus* species [45].

The differences in the locations of ribosomal RNA genes (rrnL and rrnS) (Figure 2) suggest that while their fundamental role remains unchanged, their positional variation may be linked to species-specific adaptations to parasitic lifestyles or host environments [23]. Given that *Heterodera* species parasitise a variety of host plants, it is plausible that these slight differences in mitochondrial gene arrangement relate to specific energy requirements or metabolic adjustments required for their parasitic strategies. The gene-order similarities between *H. filipjevi* and *H. schachtii* imply a more recent common ancestry, while the distinct arrangement seen in *H. cruciferae* may indicate an earlier divergence within the shared lineage (Figure 4 and Figure 5). These gene rearrangements could potentially serve as valuable phylogenetic markers [23], helping in the reconstruction of evolutionary history within the *Heterodera* genus.

The A+T content, AT skew, and GC skew were used to assess differences in base composition, providing insights into the overall nucleotide composition and potential strand asymmetry in the DNA [85]. AT skew and GC skew were specifically calculated to evaluate the nucleotide composition bias within the nematode mitogenomes. AT skew measures the difference between the frequencies of adenine (A) and thymine (T), while GC skew quantifies the difference between the frequencies of guanine (G) and cytosine (C) [63]. Analysis of nucleotide composition revealed a pronounced A+T bias in the mitogenomes of the four *Heterodera* species, with values ranging from 80.3% to 82.7%. This high A+T content, coupled with the negative AT skew and positive GC skew, is characteristic of cyst nematode mitogenomes [36,37,39,44] (Table 3). Although the skew values showed no significant differences between the *Heterodera* species, this pattern aligns with a mitochondrial genome characteristic observed in other cyst nematodes, such as those in the *Globodera* genus [36,37,38,39,40]. While most *Globodera* species exhibit negative AT skew and positive GC skew, *G. vulgaris* stands out with a positive AT skew (0.05) and negative GC skew (−0.05) [35], showing an opposing nucleotide composition trend. These differences in nucleotide composition and skew values may reflect species-specific ecological or evolutionary pressures [86]. We also speculate that environmental niche factors [87], parasitic strategies, or host–plant interactions could influence these differences in nucleotide composition [23,88].

The reconstructed maximum likelihood phylogenetic trees of both concatenated PCGs and the COX1 genes demonstrated clear clustering of major nematode groups, consistent with previous studies based on mitochondrial genome data [14]. Studies using 18S rDNA as the phylogenetic marker established the existence of three primary nematode lineages: Dorylaimia (Clade I), Enoplia (Clade II), and Chromadoria, which includes Spirurina (Clade III), Tylenchina (Clade IV), and Rhabditina (Clade V), as well as Plectida, Araeolaimida, Monhysterida, Desmodorida, and Chromadorida. Dorylaimia encompasses free-living soil nematodes, plant parasites, and vertebrate parasites such as *Trichinella* and whipworms. Enoplia is predominantly made of free-living aquatic species, with some soil nematodes and some PPNs, such as the stubby-root nematode. Chromadoria, the most diverse group, includes free-living aquatic species, notable animal parasites (e.g., *Ascaris*, hookworms, and *Dirofilaria*), plant parasites (e.g., cyst and root-knot nematodes), and the model organism *Caenorhabditis elegans* [89]. For most nematode clades, mitochondrial genome analyses have produced results that align closely with phylogenies based on nuclear gene trees, particularly those derived from SSU rDNA [14]. Mitochondrial data have frequently provided improved resolution in cases where nuclear DNA sequences were unclear or when morphological data conflicted with genetic findings [14,22]. While the 18S rRNA-based phylogeny represents the largest genetic marker for nematodes (with 2700 sequences across the entire phylum), it lacks the resolving power needed to discriminate between closely related taxa [3,14]. Although genome-based phylogenies (both nuclear and mitochondrial) hold greater potential for providing these detailed insights, they are significantly curtailed by the current limitation in taxon sampling and constraints on genomic sequencing and the assembly of good-quality draft genomes, with approximately 300 nematode nuclear genomes/transcriptomes and 250 nematode mitogenomes available [90,91,92].

The phylogenetic consistency observed between the nucleotide sequence datasets of 12 PCGs and the COX1 gene underscores the reliability of mitochondrial markers (Figure 4 and Figure 5). However, the unresolved placement of the Pratylenchidae family in the PCG dataset highlights the potential limitations of concatenated PCG data (Figure 4). In contrast, the COX1 phylogeny (Figure 5) offered a better resolution for this family, demonstrating the complementary value of single-gene and concatenated datasets in phylogenetic analyses. The relationship between *Xiphinema* species aligns with the findings of Palomares-Rius, Cantalapiedra-Navarrete et al. [50].

Certain contigs, including Contig_71773, Contig_69067, and Contig_29706, formed well-supported branches within their respective families but raised questions about their precise taxonomic placement (Figure 4 and Figure 5). For instance, Contig_71773 clustered with *Pristionchus pacificus* in the concatenated PCG analysis (Figure 4) but showed weaker support in the COX1 phylogeny (Figure 5). Similarly, while Contig_69067 and Contig_29706 were positioned within Aphelenchoididae (Figure 4 and Figure 5), it remains unclear whether they belong to the same genus or closely related genera. Several other mitochondrial contigs, such as 45034, 45035, 11153, and 19731, formed well-supported clades, yet their taxonomic identities could not be definitively resolved (Figure 4 and Figure 5). These uncertainties emphasise the need for further combined molecular and morphological studies to accurately assign these sequences to specific taxa. Future research integrating comprehensive nuclear and mitochondrial datasets, alongside robust morphological assessments, will be essential for addressing these gaps and refining nematode taxonomy and phylogeny.

Long-read sequencing offers a comprehensive genomic overview, capturing the full range of species within a sample, including those that might otherwise be missed or misidentified [78]. The incomplete nature of current nematode reference databases is a significant factor in the missed detection of certain nematode taxa, particularly when characterising economically significant or native nematode populations [93,94]. Data generated through this approach serve as a valuable resource for curating public repositories, which in turn supports more accurate and rapid species identification for biosecurity purposes. Additionally, such resources can inform the development of rapid diagnostic assays, such as loop-mediated isothermal amplification (LAMP), for deployment in the field by identifying species-specific differences for primer design [95]. While LAMP assays have been developed for *Globodera* species [96,97,98], similar rapid diagnostic tools for *Heterodera* are currently lacking [99]. Future research could explore the potential of these mitochondrial regions for creating species-specific diagnostic assays.

## 5. Conclusions

The ability to generate complete, high-quality nematode mitogenomes from a mixed pool of nematode populations holds the potential to transform biosecurity efforts in Australia and elsewhere. Mitogenome sequencing is a cost-effective sequencing strategy that has a low computational requirement for downstream analyses providing high-level taxonomic identification and valuable insights into nematode relationships. By leveraging long-read sequencing technology, Australian plant biosecurity systems can improve nematode species identification, track genetic variations, and monitor the spread of nematode populations. This approach will strengthen Australia’s capacity to quickly detect and respond to emerging biosecurity threats, ensuring more effective management of PPNs.

## Figures and Tables

**Table 1:** Mitochondrial genomes (bp), both partial and complete, and the corresponding NCBI GenBank accession numbers of mitogenomes of twenty-two plant-parasitic nematodes available on NCBI GenBank.

Family	Species	Size (bp)	NCBI GenBank Accession Number	References
Aphelenchoididae	*Aphelenchoides besseyi*	16,216	NC_025291	[41]
	*Aphelenchoides medicagus*	14,411	NC_061210	[42]
	*Bursaphelenchus mucronatus*	14,583	NC_021120	[43]
	*Bursaphelenchus xylophilus*	14,778	GQ332424	[22]
Heteroderidae	*Globodera ellingtonae*	32,122	KU726971, KU726972	[37]
	*Globodera vulgaris*	42,995	PP407208–PP407212	[35]
	*Globodera pallida*	45,071	AJ249395, DQ631911–DQ631914	[39,40]
	*Globodera rostochiensis*	41,601	EF193005, EF462976–EF462981	[36,38]
	*Punctodera chalcoensis*	6815	HM640928	[44]
	*Heterodera glycines*	14,915	HM640930	[44]
	*Heterodera cardiolata*	5295	HM640929	[44]
Meloidogynidae	*Meloidogyne arenaria*	17,580	NC_026554	[45]
	*Meloidogyne chitwoodi*	18,201	KJ476150	[46]
	*Meloidogyne oryzae*	17,066	MK507908	[47]
	*Meloidogyne graminicola*	19,589	KJ139963	[48]
	*Meloidogyne incognita*	17,662	KJ476151	[46]
	*Meloidogyne javanica*	18,921	NC_026556	[45]
Pratylenchidae	*Pratylenchus vulnus*	21,656	GQ332425	[22]
	*Radopholus similis*	16,791	FN313571	[49]
Longidoridae	*Xiphinema rivesi*	12,624	KU746820	[50]
	*Xiphinema pachtaicum*	12,489	KU746821	[50]
	*Xiphinema americanum*	12,626	NC_005928	[51]

**Table 2:** Mitochondrial genome organisation of the three *Heterodera* species, *H. filipjevi*, *H. schachtii*, and *H. cruciferae*, assembled during this study, including the type, direction, location on the strand, and size (bp) of the mitochondrial genes within each mitogenome. The protein-coding genes are highlighted in red, ribosomal RNAs are highlighted in blue, and the transfer RNAs are highlighted in green.

*Heterodera filipjevi*					*Heterodera schachtii*					*Heterodera cruciferae*				
Name	Type	Location (bp)		Size (bp)	Name	Type	Location (bp)		Size (bp)	Name	Type	Location (bp)		Size (bp)
		Minimum	Maximum				Minimum	Maximum				Minimum	Maximum	
tRNA-Ser	tRNA	1371	1432	62	ND4L	CDS	2251	2395	145	tRNA-Asp	tRNA	880	945	66
tRNA-Gln	tRNA	1706	1761	56	tRNA-Gly	tRNA	2608	2660	53	tRNA-Tyr	tRNA	1310	1364	55
tRNA-Glu	tRNA	4288	4348	61	tRNA-Asp	tRNA	7887	7946	60	ND1	CDS	1346	2314	969
tRNA-Ser2	tRNA	4540	4606	67	tRNA-Arg	tRNA	9324	9388	65	tRNA-Met	tRNA	2286	2340	55
tRNA-Leu	tRNA	5200	5263	64	rrnS	rRNA	9994	10,667	8	tRNA-Gly	tRNA	2404	2462	59
tRNA-Gly	tRNA	5605	5654	50	tRNA-Tyr	tRNA	10,737	10,792	56	rrnS	rRNA	2485	3128	644
rrnS	rRNA	5658	6333	676	ND1	CDS	10,805	11,560	756	tRNA-Glu	tRNA	3130	3187	58
ND1	CDS	6467	7309	843	ATP6	CDS	11,833	12,261	429	ATP6	CDS	3220	3753	534
tRNA-Met	tRNA	7308	7366	59	ND5	CDS	12,673	13,665	993	ND5	CDS	3869	4843	975
ATP6	CDS	7448	7924	477	tRNA-Met	tRNA	14,268	14,324	57	tRNA-Gln	tRNA	5222	5275	54
ND5	CDS	7943	9479	1537	tRNA-Ser	tRNA	14,307	14,362	56	tRNA-Ala	tRNA	5276	5323	48
tRNA-Asp	tRNA	8474	8533	60	tRNA-Cys	tRNA	14,383	14,438	56	tRNA-Leu	tRNA	5343	5398	56
tRNA-Cys	tRNA	9722	9777	56	COX2	CDS	14,519	15,094	576	COX2	CDS	5483	6146	664
COX2	CDS	9848	10,501	654	rrnL	rRNA	15,095	15,912	818	rrnL	rRNA	6306	6969	664
rrnL	rRNA	10,619	11,308	690	ND3	CDS	15,973	16,228	256	ND3	CDS	6991	7321	331
ND3	CDS	11,317	11,643	327	COX1	CDS	16,225	17,631	1407	COX1	CDS	7291	8808	1518
COX1	CDS	11,628	13,139	1512	ND2	CDS	17,882	18,358	477	ND2	CDS	8836	9505	670
ND2	CDS	13,133	13,930	798	tRNA-Ile	tRNA	18,593	18,649	57	COX3	CDS	9790	10,465	676
tRNA-Lys	tRNA	14,012	14,074	63	COX3	CDS	18,753	19,442	690	tRNA-Thr	tRNA	10,467	10,521	55
COX3	CDS	14,072	14,881	810	ND4	CDS	19,584	20,543	960	ND4	CDS	10,717	11,645	929
tRNA-Thr	tRNA	14,842	14,896	55	CYTB	CDS	20,644	21,735	1092	tRNA-His	tRNA	11,701	11,754	54
ND4	CDS	14,895	16,100	1206	tRNA-Val	tRNA	21,761	21,817	57	CYTB	CDS	11,755	12,757	1003
tRNA-His	tRNA	16,100	16,155	56	tRNA-Phe	tRNA	21,833	21,889	57	tRNA-Phe	tRNA	12,805	12,860	56
CYTB	CDS	16,207	17,173	967	ND6	CDS	21,964	22,224	261	tRNA-Ile	tRNA	13,231	13,291	61
ND6	CDS	17,421	17,918	498	tRNA-Pro	tRNA	22,880	22,932	53	tRNA-Val	tRNA	14,514	14,569	56
tRNA-Phe	tRNA	17,909	17,965	57						tRNA-Ser	tRNA	15,346	15,403	58
tRNA-Leu2	tRNA	17,976	18,034	59						tRNA-Lys	tRNA	15,974	16,044	71
tRNA-Val	tRNA	18,107	18,164	58						tRNA-Pro	tRNA	17,190	17,250	61

**Table 3:** Base contents (%) observed in the mitogenomes, along with AT skew and GC skew of the mitochondrial genomes of *Heterodera* and *Globodera* species.

Species	GenBank Accession Numbers	A%	T%	G%	C%	A+T	AT Skew	GC Skew	References
*Heterodera filipjevi*	PQ178946	25.5	54.8	12.3	7.4	80.3	−0.36	0.25	This study
*Heterodera cruciferae*	PQ325513	26.3	55.6	11.0	7.0	81.9	−0.35	0.22	This study
*Heterodera schachtii*	PQ325512	26.5	55.5	10.3	7.8	81.9	−0.35	0.14	This study
*Heterodera glycines*	HM640230	24.2	58.5	10.4	6.9	82.7	−0.41	0.20	[44]
*Globodera pallida*	AJ249395, DQ631911–DQ631914	21.9	43.7	20.6	13.8	65.6	−0.33	0.20	[39,40]
*Globodera rostochiensis*	EF193005, EF462976–EF462981	20.4	46.4	20.6	12.6	66.7	−0.39	0.24	[36,38]
*Globodera ellingtonae*	KU726971, KU726972	20.8	46.3	21.6	11.4	67.0	−0.38	0.31	[37]
*Globodera vulgaris*	PP407208–PP407212	34.8	31.2	16.2	17.9	65.9	0.05	−0.05	[35]

**Table 4:** Information on other nematode mitochondrial DNA sequences obtained from the long-read assembly of the nematode suspension, focussing on the sequence characteristics, coverage of the assembled contigs, GC contents (%), AT skew, GC skew, and similarity to known sequences submitted to NCBI GenBank based on BLASTn results.

Contig Name	Sequence Length (bp)	Average Sequencing Depth of Assembled Mitogenomes	GC%	AT Skew	GC Skew	Blastn Hit	Nucleotide BLAST % Identity
11153	14,657	×708	19.4	−0.24	0.227	*Litoditis marina*	86.41
19731	15,645	×323	21.8	−0.18	0.235	*Litoditis aff. marina PmII*	88.41
29706	15,491	×141	13.4	−0.21	0.263	*Bursaphelenchus mucronatus*	81.25
45034	13,482	×14	24.2	−0.35	0.273	*Allodiplogaster* sp.	82.98
45035	13,468	×10	24.5	−0.35	0.265	*Allodiplogaster* sp.	82.98
69067	13,103	×417	22.6	−0.27	0.23	*Panagrellus redivivus*	82.07
71552	13,227	×43	25.7	−0.5	0.37	*Toxocara vitulorum*	80.51
71773	12,498	×5	24.4	−0.16	0.205	*Pristionchus pacificus*	83.65
72261	14,950	×15	21	−0.2	0.248	*Cruznema tripartitum*	89.21

## Data Availability

The following mitochondrial sequences are deposited in NCBI GenBank: *Heterodera filipjevi* (GenBank accession number PQ178946)*, H. schachtii* (GenBank accession number PQ325512), and *H. cruciferae* (GenBank accession number PQ325513). The whole-genome shotgun sequencing project was accessed from DDBJ/ENA/GenBank under BioProject PRJNA1109461. Raw reads from the long-read assembly can be made available upon request to the authors.

## Supplementary Materials

The following supporting information can be downloaded at https://www.mdpi.com/article/10.3390/pathogens14030234/s1. Table S1. Maximum likelihood tree was inferred from nucleotide sequences of the twelve concatenated mitochondrial protein-coding genes, including ND1, ND2, ND3, ND4, ND5, and ND6 (NADH dehydrogenase subunits 1–6); COX1, COX2, and COX3 (cytochrome c oxidase subunits 1–3); CYTB (cytochrome b); ND4L (subunit 4L of NADH dehydrogenase); and ATP6 (Adenosine triphosphate subunit 6) across 44 nematode mitogenomes, using MAFFT alignment of PCG sequences under the GTR Gamma I model in RAxML. The accession numbers of the nematode species along with their mitochondrial genome sizes (bp), concatenated PCG sequences sizes (bp), and mitochondrial organisation and references are reported. Table S2. Maximum likelihood tree was inferred from nucleotide sequences of the COX1 gene across 45 nematode mitogenomes, using MAFFT alignment of COX1 sequences under the GTR Gamma I model in RAxML. The accession numbers of the nematode species along with their mitochondrial genome sizes (bp) and COX1 gene sizes (bp) and their mitochondrial organisation and references are reported. References [100,101,102,103,104,105] are cited in the Supplementary Materials.

## Abbreviations

The following abbreviations are used in this manuscript:

PPN	Plant-parasitic nematode
HTS	High-throughput sequencing
NCBI	National Center for Biotechnology Information
BLAST	Basic Local Alignment Search Tool
ND	NADH dehydrogenase
ND4L	Subunit 4L of NADH dehydrogenase
ATP	Adenosine triphosphate subunit
COX	Cytochrome c oxidase subunit
CYTB	Cytochrome b
rRNA	Ribosomal RNA
rrnL	16S ribosomal RNA
rrnS	12S ribosomal RNA
tRNA	Transfer RNA
mtDNA	Mitochondrial DNA
nDNA	Nuclear DNA
ONT	Oxford Nanopore Technology
LAMP	Loop-mediated isothermal amplification
ML	Maximum likelihood
