# Supplementary material for: High-Throughput Sequencing Enables Rapid Analyses of Nematode Mitochondrial Genomes from an Environmental Sample
Source: Pathogens. 2025 Feb 27;14(3):234. doi: 10.3390/pathogens14030234 (PMC11944570; doi:10.3390/pathogens14030234)
Supplement: Supplementary file 1 [file pathogens-14-00234-s001.zip › pathogens-3480460-supplementary.pdf]

Supplementary Table S1 Maximum likelihood tree was inferred from nucleotide sequences of the twelve concatenated mitochondrial protein-coding genes including ND1, ND2, ND3, ND4, ND5 and ND6 (NADH dehydrogenase subunits 1–6), COX1, COX2 and COX3 (cytochrome c oxidase subunits 1–3), CYTB (cytochrome b), ND4L (subunit 4L of NADH dehydrogenase), and ATP6 (Adenosine triphosphate subunit 6) across 44 nematode mitogenomes, using MAFFT alignment of PCG sequences under the GTR Gamma I model in RAxML. The accession numbers of the nematode species along with their mitochondrial genome size (bp), concatenated PCG sequences size (bp), their mitochondrial organisation and references are reported.

**Accession number**	**Species**	**Mitochondrial genome size (bp)**	**Complete / Incomplete**	**Concatenated PCG size (bp)**	**References**
NC_025291	*Aphelenchoides besseyi*	16,216	Complete	10,125	[41]
NC_061210	*Aphelenchoides medicagus*	14,411	Complete	10,172	[42]
NC_021120	*Bursaphelenchus mucronatus*	14,583	Complete	10,222	[43]
NC_023208	*Bursaphelenchus xylophilus*	14,778	Complete	10,214	[43]
NC_036308	*Camallanus cotti*	17,901	Complete	11,060	[21]
NC072121	*Cruznema tripartitum*	14,067	Complete	10,293	[71]
KU726971, KU726972	*Globodera ellingtonae*	32,122	Complete	9,934	[37]
AJ249395, DQ631911–DQ631914	*Globodera pallida*	45,071	Complete	6,595	[40], [39]
EF193005, EF462976–EF462981	*Globodera rostochiensis*	41,601	Complete	2,238	[36], [38]
PP407208–PP407212	*Globodera vulgaris*	42,995	Complete	9,649	[35]
HM640930	*Heterodera glycines*	14,915	Partial	9,909	[44]
NC_008534	*Heterorhabditis bacteriophora*	18,128	Complete	10,304	[100]
NC_033867	*Longidorus vineacola*	13,519	Complete	10,114	[50]
NC_026554	*Meloidogyne arenaria*	17,580	Complete	9,791	[45]
KJ476150	*Meloidogyne chitwoodi*	18,201	Complete	9,774	[46]
NC_026555	*Meloidogyne enterolobii*	17,053	Complete	9,789	[45]
NC_056772	*Meloidogyne graminicola*	19,589	Complete	9,879	[48]

KJ476151	*Meloidogyne incognita*	17,662	Complete	9,794	[46]
NC_026556	*Meloidogyne javanica*	18,291	Complete	9,794	[45]
MK507908	*Meloidogyne oryzae*	17,066	Complete	9,719	[47]
NC_033868	*Paralongidorus litoralis*	12,763	Complete	10,002	[50]
NC_020434	*Pratylenchus vulnus*	21,656	Complete	10,038	[22]
NC_015245	*Pristionchus pacificus*	15,954	Complete	10,299	[101]
NC_013253	*Radopholus similis*	16,791	Complete	10,128	[49]
NC_013827	*Teladorsagia circumcincta*	14,066	Complete	10,264	[102]
NC_070176	*Toxocara vitulorum*	15,045	Complete	10,292	[103]
NC_028621	*Trichuris muris*	14,105	Complete	10,699	[104]
JQ996232	*Trichuris ovis*	13,946	Complete	10,773	[33]
GU070737	*Trichuris suis*	14,436	Complete	10,920	[105]
NC_005928	*Xiphinema americanum*	12,626	Complete	10,104	[51]
KU746821	*Xiphinema pachtaicum*	12,489	Complete	9,921	[50]
KU746820	*Xiphinema rivesi*	12,624	Complete	10,116	[50]

Supplementary Table S2 Maximum likelihood tree was inferred from nucleotide sequences of the COX1 gene across 45 nematode mitogenomes, using MAFFT alignment of COX1 sequences under the GTR Gamma I model in RAxML. The accession numbers of the nematode species along with their mitochondrial genome size (bp) and the COX1 gene size (bp), their mitochondrial organisation and references are reported.

Accession number	Species	Mitochondrial genome size (bp)	Complete / Incomplete	COX1 gene size (bp)	References
NC_025291	*Aphelenchoides besseyi*	16,216	Complete	1,575	[41]
NC_061210	*Aphelenchoides medicagus*	14,411	Complete	1,581	[42]
NC_021120	*Bursaphelenchus mucronatus*	14,583	Complete	1,563	[43]
NC_023208	*Bursaphelenchus xylophilus*	14,778	Complete	1,563	[43]
NC_036308	*Camallanus cotti*	17,901	Complete	1,554	[21]
NC072121	*Cruznema tripartitum*	14,067	Complete	1,575	[71]
KU726971, KU726972	*Globodera ellingtonae*	32,122	Complete	1,509	[37]
AJ249395, DQ631911–DQ631914	*Globodera pallida*	45,071	Complete	1,389	[40], [39]
EF193005, EF462976–EF462981	*Globodera rostochiensis*	41,601	Complete	1,389	[36], [38]
PP407208–PP407212	*Globodera vulgaris*	42,995	Complete	1,582	[35]
HM640930	*Heterodera glycines*	14,915	Partial	1,524	[44]
HM640929	*Heterodera cardiolata*	5,295	Partial	1,533	[44]
NC_008534	*Heterorhabditis bacteriophora*	18,128	Complete	1,587	[100]
NC_033867	*Longidorus vineacola*	13,519	Complete	1,548	[50]
NC_026554	*Meloidogyne arenaria*	17,580	Complete	1,522	[45]
KJ476150	*Meloidogyne chitwoodi*	18,201	Complete	1,521	[46]
NC_026555	*Meloidogyne enterolobii*	17,053	Complete	1,522	[45]
NC_056772	*Meloidogyne graminicola*	19,589	Complete	1,554	[48]
KJ476151	*Meloidogyne incognita*	17,662	Complete	1,522	[46]
NC_026556	*Meloidogyne javanica*	18,291	Complete	1,522	[45]

MK507908	*Meloidogyne oryzae*	17,066	Complete	1,513	[47]
NC_033868	*Paralongidorus litoralis*	12,763	Complete	1,539	[50]
NC_020434	*Pratylenchus vulnus*	21,656	Complete	1,533	[22]
NC_015245	*Pristionchus pacificus*	15,954	Complete	1,578	[101]
NC_013253	*Radopholus similis*	16,791	Complete	1,557	[49]
NC_013827	*Teladorsagia circumcincta*	14,066	Complete	1,578	[102]
NC_070176	*Toxocara vitulorum*	15,045	Complete	1,581	[103]
NC_028621	*Trichuris muris*	14,105	Complete	1,545	[104]
JQ996232	*Trichuris ovis*	13,946	Complete	1,545	[33]
GU070737	*Trichuris suis*	14,436	Complete	1,542	[105]
NC_005928	*Xiphinema americanum*	12,626	Complete	1,552	[51]
KU746821	*Xiphinema pachtaicum*	12,489	Complete	1,542	[50]
KU746820	*Xiphinema rivesi*	12,624	Complete	1,548	[50]
